# Incident Tuberculosis during Antiretroviral Therapy Contributes to Suboptimal Immune Reconstitution in a Large Urban HIV Clinic in Sub-Saharan Africa

**DOI:** 10.1371/journal.pone.0010527

**Published:** 2010-05-07

**Authors:** Sabine M. Hermans, Agnes N. Kiragga, Petra Schaefer, Andrew Kambugu, Andy I. M. Hoepelman, Yukari C. Manabe

**Affiliations:** 1 Department of Internal Medicine and Infectious Diseases, University Medical Center Utrecht, Utrecht, The Netherlands; 2 Infectious Diseases Institute, Makerere University College of Health Sciences, Kampala, Uganda; 3 Division of Infectious Diseases, Department of Medicine, Johns Hopkins University School of Medicine, Baltimore, Maryland, United States of America; University of Cape Town, South Africa

## Abstract

**Background:**

Antiretroviral therapy (ART) effectively decreases tuberculosis (TB) incidence long-term, but is associated with high TB incidence rates in the first 6 months. We sought to determine the incidence and the long-term effects of TB during ART on HIV treatment outcome, and the risk factors for incident TB during ART in a large urban HIV clinic in Uganda.

**Methodology/Principal Findings:**

Routinely collected longitudinal clinical data from all patients initiated on first-line ART was retrospectively analysed. 5,982 patients were included with a median baseline CD4+ T cell count (CD4 count) of 117 cells/mm^3^ (interquartile range [IQR]; 42, 182). In the first 2 years, there were 336 (5.6%) incident TB events in 10,710 person-years (py) of follow-up (3.14 cases/100pyar [95% CI 2.82–3.49]); incidence rates at 0–3, 3–6, 6–12 and 12–24 months were 11.25 (9.58–13.21), 6.27 (4.99–7.87), 2.47 (1.87–3.36) and 1.02 (0.80–1.31), respectively. Incident TB during ART was independently associated with baseline CD4 count of <50 cells/mm^3^ (hazard ratio [HR] 1.84 [1.25–2.70], *P* = 0.002) and male gender (HR 1.68 [1.34–2.11], *P*<0.001). After two years on ART, the patients who had developed TB in the first 12 months had a significantly lower median CD4 count increase (184 cells/mm^3^ [IQR; 107, 258, n = 118] vs 209 cells/mm^3^ [124, 309, n = 2166], *P* = 0.01), a larger proportion of suboptimal immune reconstitution according to two definitions (increase in CD4 count <200 cells/mm^3^: 57.4% vs 46.9%, *P* = 0.03, and absolute CD4 count <200 cells/mm^3^: 30.4 vs 19.9%, *P* = 0.006), and a higher percentage of immunological failure according to the WHO criteria (13.6% vs 6.5%, *P* = 0.003). Incident TB during ART was independently associated with poor CD4 count recovery and fulfilling WHO immunogical failure definitions.

**Conclusions/Significance:**

Incident TB during ART occurs most often within 3 months and in patients with CD4 counts less than 50 cells/mm^3^. Incident TB during ART is associated with long-term impairment in immune recovery.

## Introduction

The use of antiretroviral therapy (ART) causes major reductions in HIV-associated morbidity and mortality, and decreases the risk of developing tuberculosis (TB) by 70–90% [1]–[4]. This reduction is time-dependent, however. Progressive immune reconstitution decreases the risk of TB in the long-term, but there are high incidence rates of TB early after ART initiation both in developed countries and in resource-limited settings [5]. Incident TB during ART is often due to “unmasking” of reactivated TB because of restoration of TB antigen-specific functional immune responses. A subset of these cases will have inflammatory symptoms consistent with immune reconstitution inflammatory syndrome (IRIS) [6]. The high incidence of TB early during ART significantly contributes to the high mortality rates early after ART initiation reported in resource-limited settings [7]–[9]. Patients with very low baseline CD4+ T cell counts (CD4 counts) are at higher risk for unmasking TB as well as for dying early after ART initiation. They are also more likely to have suboptimal immunological recovery, which has been reported in both resource-rich and resource-limited settings [10]–[15].

Suboptimal immune reconstitution affects up to 30% of all patients initiated on ART [10], [16]. Definitions vary, but immunological non-responders in developed countries are, in general, identified by a <30% increase in CD4 count or an absolute CD4 count <200 cells/mm^3^ with full suppression of HIV replication during the first 6–12 months of ART [10]. Current CD4 counts are the most important determinant of mortality after ART initiation [13], [17], [18]. In developed countries, immunological non-responders also have an approximately doubled relative risk of clinical progression to AIDS compared to patients with an adequate response [10], although a study conducted in our own clinic could not confirm these findings in a resource-limited setting [11]. In addition, there was a recent report from Spain that immunological non-responders also have a higher rate of non-AIDS related mortality [19].

We sought to determine the incidence of, the risk factors for and the long-term effects of incident TB on HIV treatment outcome in a large urban HIV clinic in Uganda.

## Methods

### Setting

The Adult Infectious Diseases Clinic (AIDC) at the Infectious Diseases Institute (IDI), at the Makerere University College of Health Sciences in Kampala, provides outpatient care to over 20,000 registered HIV-positive patients since its inception in 2002. Currently more than 10,000 patients are in active follow-up, of whom over 8,000 have been initiated on ART. Treatment is based on the national ART guidelines of the Ugandan Ministry of Health, which, at the time of our study, consisted of daily co-trimoxazole prophylaxis for all patients irrespective of CD4 count, and ART initiation in those with a prior AIDS diagnosis (WHO stage IV disease) or a CD4 count <200 cells/mm^3^ [20]. First-line ART comprised of stavudine (d4T) or zidovudine (AZT) in combination with lamivudine (3TC) plus a non-nucleoside reverse transcriptase inhibitor in standard doses (nevirapine (NVP) or efavirenz (EFV)). Choice of ART is at the physician's discretion and is also dependent on availability of the different first line options. Clinical screening for active opportunistic infections including TB takes place prior to ART initiation. Available investigations for TB include fluorescence sputum microscopy, chest radiology, abdominal ultrasonography, and fine-needle aspiration of lymphadenopathy for acid fast bacilli microscopy and cytology. Diagnosis of TB is made on the basis of these investigations, but very often on clinical grounds. No mycobacterial culture facilities are available for routine evaluation. Patients diagnosed with active TB are treated with a standard 8 month regimen with a 8 week, 4 drug (isoniazid, rifampicin, ethambutol and pyrazinamide) intensive phase and a subsequent 6 month, 2 drug (isoniazid, ethambutol) continuation phase. Scheduled clinic appointments take place every 4 weeks with monitoring of clinical status and adherence, CD4 counts are performed every 6 months by FACS calibur (Becton Dickinson). Viral load monitoring (Roche Amplicor, with a detection limit of 400 copies/mm^3^) is not routine and is only available for patients suspected of virological failure on clinical and immunological grounds. Patients requiring inpatient care are referred to Mulago National Referral Hospital, a tertiary care hospital in the same complex. All treatment at the IDI is free of charge.

### Data collection and ethics statement

Data on clinical parameters, ART and adherence, WHO stage, toxicities and opportunistic infections is routinely collected into a database, to which laboratory data is added. A separate database has recently been populated with pharmacy data on TB drug prescriptions since 2006. A team of trained nurses and medical officers verify data with the patient's medical notes and audit this database as part of regular clinic monitoring and evaluation. Use of this database for clinical research has been approved by the Institutional Review Boards (IRB) of IDI, Makerere University and the Uganda National Council for Science and Technology. The data for our study was extracted from this database and analysed anonymously.

### Study design and selection criteria

Incident TB (defined by the start of antituberculous therapy) in the first 2 years after ART initiation was ascertained retrospectively in all patients started on ART at IDI from January 2003 to January 2009. Patients who initiated ART elsewhere were excluded as well as patients initiated on second-line ART. Patients with a history of active TB were excluded from the analysis (Figure S1 and Table S1). The TB drug prescription database was merged with the clinic database to validate the coding of new tuberculosis after ART initiation (Figure S2). Patients coded as having developed TB after ART initiation in both databases were considered as TB cases. The TB status of cases identified in only one of the two databases was ascertained by the validation team after review of charts, confirming TB diagnosis by a clinician and starting date of ART and TB treatment. This included all cases of TB before initiation of the TB drug prescription database in 2006. Patients whose charts were unavailable for review were excluded from the analysis. Patients who were treated for TB after first-line ART initiation were considered TB cases in the analysis, whether they were diagnosed in our clinic or elsewhere, and whether the diagnosis was based on bacteriological testing or clinical suspicion.

### Definitions

Loss to follow-up after ART initiation was defined as no documented clinic attendance for more than 90 days [21]. Immunological failure at 24 months was defined by fulfilment of one or more of the World Health Organization (WHO) criteria: decrease in CD4 count to pre-ART level or below, decrease in CD4 count from on-treatment peak value by more than 50% or persistent CD4 count <100 cells/mm^3^ [22]. For suboptimal immune response at 24 months on ART, we used two previously used definitions of a CD4 count increase of <200 cells/mm^3^ and not attaining an absolute CD4 count >200 cells/mm^3^ [11]. To determine effects of TB on CD4 count, suboptimal immune response and immunological failure at 24 months, we restricted our analysis to patients who developed TB within 12 months after ART initiation and to those who remained TB free during the entire 24 months of follow-up to exclude patients on treatment for active TB at the time that CD4+ T cell recovery was compared.

### Statistical methods

TB incidence rates were calculated per 100 person years at risk (pyar). Patients were censored at the time of TB diagnosis or at the date of death, transfer or, in the case of loss-to-follow-up, date of last visit, or the last visit date before January 2009. Risk factors associated with the incidence of TB were analysed using a Cox proportional hazards model. Differences in the median absolute and median changes in CD4 counts and CD4 percentages at 24 months were compared between patients who never had TB and those who did using the Mann-Whitney test, while the proportions of immunological failure and suboptimal immune reconstitution were compared between these two groups using chi-square tests. Multiple linear regression models were fitted to investigate the effect of incident TB during ART on CD4 count (absolute and change from baseline) adjusting for baseline CD4 count, sex, AZT-based regimen and age. For this, generalised estimating equations were used to account for the intra-individual correlation of repeated measurements. Robust standard errors and an exchangeable correlation matrix with a linear link were employed. Multivariable logistic regression analysis was performed to investigate the effect of incident TB on immunological failure adjusting for the same factors.

In all regression models risk factors were explored using univariable regression analysis and were retained in the multivariable model based on forward selection; hypothesized risk factors were included and confounders were retained based on univariable *P*<0.2. Interactions between variables were investigated and tested using the likelihood ratio test, and were included in the model if significant. All statistical tests were two-sided at an α value of 0.05 and were conducted using STATA version IC 10.0 (College Station, Texas, USA).

## Results

### Patient characteristics and follow-up

A total of 5982 HIV-infected adults started on first line ART between January 2003 and January 2009 were included. Baseline characteristics of the included patients are shown in Table 1. The majority of the patients were women (66.5%). The mean age of the cohort was 37.2 years and the median baseline CD4 count was 117 cells/mm^3^ (interquartile range [IQR]; 42, 182). Data on baseline CD4 count and CD4 percentage were not available for 282 patients (16 of whom had incident TB). 96.8% of patients were started on two nucleosides (NRTI) and a non-nucleoside reverse transcriptase inhibitor (NNRTI).

**Table 1:** Baseline characteristics.

Category	Subcategory	All patients (5982 [100%])	TB cases (N = 336 [5.6%])	Non-TB cases (N = 5646 [94.3%])
**Sex** (female)		3978 (66.5)	185 (55.1)	3793 (67.2)
**Age** (years, mean [SD])		37.2 (8.7)	36.9 (8.7)	37.2 (8.7)
**WHO stage** a	I&II	2210 (37.1)	98 (29.2)	2112 (37.6)
	III	2367 (39.7)	145 (43.2)	2222 (39.5)
	IV	1380 (23.2)	93 (27.7)	1287 (22.9)
**Baseline CD4 count** a (cells/mm^3^, median [IQR])		117 (42, 182)	85.5 (31.5, 153.5)	119 (43, 183)
**Baseline CD4 count** a (cells/mm^3^)	≥200	988 (17.3)	36 (11.3)	952 (17.7)
	50–199	3140 (55.1)	172 (53.8)	2968 (55.2)
	<50	1572 (27.6)	112 (35.0)	1460 (27.1)
**Baseline CD4 percentage** a (median [IQR])		7.0 (3.3, 11.4)	6.0 (3.0, 9.0)	7.0 (3.4, 11.8)
**ART regimen**	d4T+3TC+NVP	3205 (53.6)	215 (64.0)	2990 (53.0)
	d4T+3TC+EFV	75 (1.3)	9 (2.7)	66 (1.2)
	AZT+3TC+NVP	351 (5.9)	11 (3.3)	340 (6.0)
	AZT+3TC+EFV	2159 (36.1)	94 (28.0)	2065 (36.6)
	Other 1st line b	192 (3.2)	7 (2.1)	185 (3.3)

TB, tuberculosis; ART, antiretroviral therapy; IQR, interquartile range; d4T, stavudine; 3TC, lamivudine; NVP, nevirapine; AZT, zidovudine; EFV, efavirenz.

a Data on CD4 count and CD4 percentage were not available for 282 patients (16 with incident TB); CD4 counts and percentages were closest recorded values to the baseline start date, maximum 6 months pre and 15 days post ART initiation. Data on WHO stage were not available for 25 patients.

b Other first line triple ART regimens.

### Incident TB and risk factors

**Figure 1:**
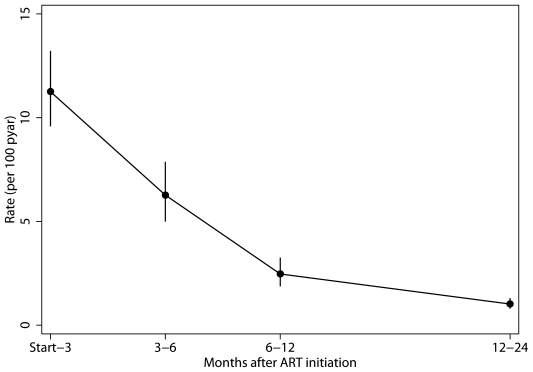
Tuberculosis incidence rates after ART initiation. TB incidence rates according to time of TB occurrence post-ART. Point estimates of incidence rates (cases/100pyar, [95% CI]): 0–3 months: 11.25 (9.58–13.21), 3–6 months: 6.27 (4.99–7.87), 6–12 months: 2.47 (1.87–3.36) and 12–24 months: 1.02 (0.80–1.31). (ART, antiretroviral therapy; TB, tuberculosis; CI, 95% confidence interval; pyar, person years at risk.)

In the first two years of ART, there were 336 (5.6%) new TB events in 10,710 person-years of follow-up (3.14 cases/100pyar [95% CI 2.82–3.49]). Incidence rates of TB at 0–3, 3–6, 6–12 and 12–24 months were 11.25 (9.58–13.21), 6.27 (4.99–7.87), 2.47 (1.87–3.36) and 1.02 (0.80–1.31) cases/100pyar, respectively (Figure 1). The multivariable Cox proportional hazards analysis showed that a baseline CD4 count of <50 cells/mm^3^ (hazard ratio [HR] 1.84 [1.25–2.70], *P* = 0.002) and male gender (HR 1.68 [1.34–2.11], *P*<0.001) were significantly associated with an increased risk of TB. Age per 10 years' rise (HR 0.91 [0.79–1.04], *P = *0.17) and year of ART initiation (HR 1.03 [0.93–1.14], *P* = 0.55) were not associated. Hazard ratios were similar when the analysis was done using baseline CD4 count quartiles or including only patients with complete baseline CD4 count data (data not shown).

### Association between incident TB and immunological response to ART

**Figure 2:**
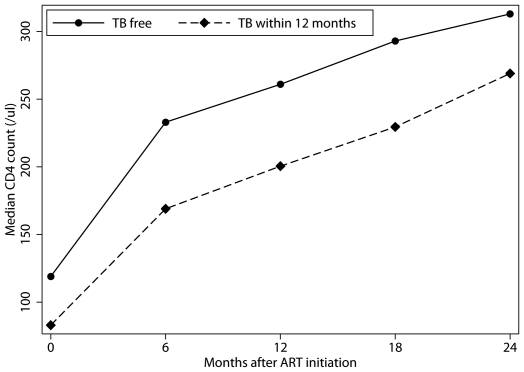
Trajectories of median CD4 counts in the first two years of ART by TB status. Patients with incident TB during ART (triangles with dashed line) compared to patients who remained TB free (circles with solid line) throughout the follow-up period. (ART, antiretroviral treatment; TB, tuberculosis).

CD4 count (absolute and percentage) responses to ART in TB cases were determined and compared with the responses observed among those who remained free of TB. 2376 subjects with 24 months of follow-up data were available for analysis, of whom 123 (5.1%) had developed TB in the first 12 months on ART and 2209 (93.0%) who had remained TB-free after 2 years on ART. 44 (1.9%) patients developed TB between 12 and 24 months on ART, these were excluded from the analysis. Data on their HIV treatment outcome is summarised in Table S2. At two years, both the median absolute CD4 count (269 cells/mm^3^ [IQR; 179, 363] vs 312 cells/mm^3^ [221, 431], *P* = 0.002) and the median CD4 count increase (184 cells/mm^3^ [107, 258] vs 208 cells/mm^3^ [123, 309], *P* = 0.02) were significantly lower in the TB group. There was no significant difference in median absolute or median change in CD4 percentages: 17 (12, 22) vs 17 (13, 22), *P* = 0.20 and 12 (7, 15) vs 10 (7, 14), *P* = 0.51. The incident TB group had a larger proportion of suboptimal immune reconstitution according to both definitions: a CD4 count increase of <200 cells/mm^3^ after 24 months on ART (56.8% vs 47.1%, *P* = 0.005) and not attaining an absolute CD4 count >200 cells/mm^3^ (30.1% vs 20.1%, *P*<0.001). Multiple linear regression analysis using generalised estimating equations of the factors associated with CD4 change at two years confirmed that incident TB during ART was associated with lower CD4 count recovery (β-coefficient [95% CI]; −143.05 [−191.47–94.64], *P*<0.001). Poor CD4 count restoration was also associated with male sex (Table 2). Higher baseline CD4 counts were associated with a lower increase at two years. Effect modification between sex and baseline CD4 count was found, which could be explained by a lower median baseline CD4 count in men. Age and the use of an AZT-based regimen were not associated with CD4 count change at 2 years. Results of multiple linear regression analysis of the association of these factors with absolute CD4 counts at 24 months were similar (data not shown), with the exception of a positive effect of a higher baseline CD4 count which was to be expected. Trajectories of median CD4 counts over time in the two groups are shown in Figure 2.

**Table 2:** Multiple linear regression of the relationship with CD4 change after 24 months of ART.

	Subcategory	β-Coefficient (95% CI)	*P*-value
**Incident TB after ART**	No	1	
	Yes	−143.05 (−191.47–94.64)	**<0.001**
**Baseline CD4 count** (cells/mm^3^, per 50 cells' rise)		−23.38 (−32.99–13.78)	**<0.001**
**Sex**	Female	1	
	Male	−48.04 (−63.03–33.05)	**<0.001**
**Interaction term baseline CD4 and sex**		0.17 (0.05–0.29)	**0.004**
**Age** (years, per 10 years' rise)		−1.56 (−5.92–2.79)	0.48
**Regimen**	Non-AZT based	1	
	AZT-based	4.56 (−1.64–10.76)	0.15

ART, antiretroviral treatment; TB, tuberculosis; CI, confidence interval; AZT, zidovudine.

Furthermore, a higher proportion of patients who developed TB in the first 12 months after ART initiation could be classified as immunological failure cases at 24 months according to the WHO criteria than those who did not (13.0% vs 7.5%, *P* = 0.001). This effect was confirmed by multivariable logistic regression analysis which showed a significantly increased likelihood of immunological failure (odds ratio [OR]; 2.02 [1.13–3.59], *P* = 0.02) with incident TB, independent of baseline CD4 count, male sex, older age and the use of AZT (Table 3).

**Table 3:** Multivariable logistic regression of immunological failure after 24 months on ART.

	Odds Ratio (95% CI)	*P*-value
**Incident TB after ART**	2.02 (1.13–3.59)	**0.02**
**Baseline CD4 count** (cells/mm^3^, per 50 cells' rise)	1.22 (1.14–1.30)	**<0.001**
**Male sex**	1.30 (0.93–1.82)	0.13
**Age** (years, per 10 years' rise)	0.89 (0.73–1.08)	0.25
**AZT-based regimen**	1.61 (1.14–2.27)	**0.007**

ART, antiretroviral treatment; TB, tuberculosis; CI, confidence interval; AZT, zidovudine.

### Loss to follow-up and mortality

After two years on ART, 888 (14.8%) patients were lost to follow-up and 596 (10.0%) had been transferred to a clinic elsewhere. The median follow-up time was 603 days (IQR 196, 708). 363 (6.1%) patients died in the first two years of ART, with a rate of 3.01 per 100 pyar (95% CI 2.71–3.35). Mortality rates for 0–3, 3–6, 6–12 and 12–24 months mirrored TB incidence rates: 10.05 (8.49–11.89), 4.76 (3.68–6.15), 2.89 (2.25–3.71) and 1.30 (1.05–1.61), respectively (Figure S3).

## Discussion

Patients who developed TB after ART initiation in our large urban ART cohort were more likely to have suboptimal immune reconstitution despite TB treatment. Active TB in the first year after ART initiation had long-lasting effects on immune restoration: the median CD4 count change at 2 years was significantly lower after correction for lower baseline CD4 count. These patients also fulfilled the WHO criteria for immunological failure more often. To our knowledge, the immunosuppressive effect of incident active TB after ART initiation has not been previously described.

The potential of virological and immunological response to ART seems to be equal in both resource-rich and resource-limited settings [12], [16], [23], [24], although there are some reports that less overall immune recovery occurs more often in resource limited settings [14], [23]. The most important factor associated with suboptimal immune reconstitution in all settings is baseline CD4 count, but other factors have also shown to be associated, such as higher baseline HIV viral loads, male sex, older age and hepatitis C co-infection [10], [16], [25]–[30]. The use of AZT-based regimens has also been implicated [11], [31], [32]. We were able to analyse and identify an association with most of these factors in our study, except for viral load and hepatitis C co-infection as our setting does not allow for routine viral load and serologic testing. A higher baseline CD4 count was associated with a lower CD4 increase after two years of ART, which is consistent with previous reports that show that patients with the lowest baseline CD4 count tend to have the largest increase [33], [34]. In multiple linear regression analysis, the negative effect of incident TB during ART on immune restoration was independently associated.

The underlying mechanisms of immunological non-response are not completely understood, but it seems that a complex model of immune activation, T cell turnover and homeostatic regulation is responsible for CD4+ T cell loss [10]. Excessive T cell destruction due to T cell activation plays an important role, and has been shown to persist even after virological suppression occurs [35]–[37]. Higher levels of T cell activation have been reported in HIV-seronegative Africans [38], and have been attributed to frequent infections by various endemic pathogens, such as parasitic infections. It has been suggested that co-infections might limit the capacity for restoration of CD4 counts through an immunosuppressive effect on hematopoiesis and, most importantly, through augmentation of the HIV-induced heightened immune activation leading to widespread apoptosis of both HIV-infected and uninfected lymphocytes [39], [40]. In a small study done in Uganda, the presence of co-infection with highly prevalent and endemic pathogens in patients with and without ART was associated with increased CD4+ T cell activation independent of CD4 count and viral load [41]. Moreover, in another study done also in Uganda, patients who developed an AIDS-defining condition according to the WHO clinical staging system within 12 weeks pre or post ART initiation, took longer to achieve an increase in CD4 count of 50 cells/mm^3^ [42].

TB induced immunosuppression is a well-studied area: an investigation of T cell cytokine responses in HIV-negative pulmonary TB patients showed a persistent depressed tuberculin-induced IFN-γ response up to 18 months despite successful treatment, suggesting a long-lasting depletion or primary dysfunction of antigen-responsive T cells from the peripheral blood due to active TB [43]. Furthermore, apoptosis of *Mycobacterium tuberculosis*-reactive CD4+ and non-CD4+ T cells seems to be increased in pulmonary TB patients [44]. Taken together, these results suggest that TB may have a long-lasting immunosuppressive effect, which could account for the persistent decreased CD4 count levels we found in our patients with incident TB. We hypothesize that the co-infection leads to a long-lasting augmentation of already heightened T cell activation in HIV-infected patients, followed by increased apoptosis of CD4 cells with decreased immune restoration.

Our data suggests that there may be a specific effect of incident TB after ART initiation on immune recovery. A Malawian study of 76 clinical ART failure cases showed that extrapulmonary TB and Kaposi's sarcoma were the most common conditions causing misclassification compared to virologic failure gold standard, but these factors were not significant in multivariable analysis [45]. Other studies have reported that AIDS events (including TB) before or at the time of ART initiation are not associated with decreased immune recovery [46]–[48], although the numbers of patients analysed were small and this was not their primary study objective. There has also been a report suggesting the contrary [14]. In a study from South Africa, there was no effect on immune recovery with prevalent and early incident TB together, although a relatively low proportion (25%) of patients were analysable at 48 weeks of follow-up [49].

A recent paper published by Lawn and colleagues showed that the time spent at lower CD4 count levels after ART initiation was the predominant predictor of TB risk at 4 months of ART and onwards in South Africa [50]. To address this issue in our cohort, we restricted our analysis at 2 years after ART initiation to compare CD4 counts of patients who initiated TB treatment within the first 12 months of ART, and excluded all cases of active (and thus also recurrent) TB. Therefore, our results do not examine the duration of the effect of incident TB on immune recovery. The poor CD4+ T cell reconstitution could also represent a temporary delay in immune recovery followed by the same recovery rate as non-TB patients as suggested by the similarity in slope in figure 2. Patients with incident TB after ART would still spend a significantly longer amount of time at lower CD4 counts compared to patients who were TB free after ART.

As TB is the most frequent opportunistic infection in HIV-infected patients in our setting, it is possible that the decreased CD4 counts in patients after TB treatment may partly account for the high rates of misclassification of treatment failure based on the WHO immunological criteria, which have been reported in a number of recent articles from sub-Saharan Africa [45], [51]–[56]. The sensitivities of the WHO criteria ranged from 8% to 23% with specificities from 90% to 98%. In our study, patients with incident TB during ART were twice as likely to be classified as having immunological failure after 2 years on ART than those without, a finding which was confirmed in multivariable logistic regression to be independent of other associated factors. Misclassification of these patients as failing immunologically puts them at risk for inappropriate switching to second line therapy. The immunosuppressive effect was not seen with CD4 count percentages. The CD4 percent may be a better parameter to use in settings without access to viral load confirmation of failure, although this data highlights the need for more affordable and accessible viral load testing [51]–[53], [55].

Our large retrospective cohort study also reported comparable incidence rates of ART-associated TB to those published from earlier work in high-endemic areas [5], [57]–[59]. As expected, incident TB during ART was a common occurrence in our study. Incidence rates were very high in the first three months, and decreased to a level which was still almost three fold higher than in the general Ugandan population [60]. Mortality rates after ART initiation mirrored these rates. Identified risk factors for developing TB after ART initiation in our cohort were a low baseline CD4 count and male sex (both have been previously described [5], [57]–[59], [61]) and highlight the importance of initiating ART earlier.

Our study had several limitations. Because of the retrospective design the data was often incomplete, although we validated our data with chart reviews. We excluded a large number of possible TB cases that could not be verified by chart review which may have introduced bias. Although our population was large, the presented data was collected at only one centre, possibly affecting the generalisability of our results. We are also aware of the possible under- and over-diagnosis of TB in our setting since mycobacterial culture is not routinely used to confirm cases of smear-negative TB which are frequent in HIV-positive populations. We do feel that our setting reflects the current practice in many clinics in sub-Saharan Africa. Finally, an alternative explanation for the lower CD4+ T cell count levels in patients with incident TB during ART is decreased adherence to ART during TB treatment due to high pill burden, and side-effects [32]. This may have lead to lower adherence levels and thus slower immune recovery. Insensitive measurements of adherence in our clinic (self-report and pill counts) in combination with infrequent viral load testing made it difficult to exclude this possibility. Nonetheless, the majority of the TB patients who developed suboptimal immune reconstitution for whom a viral load was measured (6 of 8 patients) had an undetectable viral load.

In conclusion, in this large urban HIV clinic in Uganda the incidence of TB is very high in the first three months after ART initiation, especially in patients with very low CD4 counts, and likely contributes to high early mortality. Furthermore, incident TB after ART may have a long-lasting effect on immune recovery which, in turn, is associated with a higher risk of opportunistic infections, mortality and non-infectious complications of AIDS. The biologic importance of our finding of suboptimal CD4 recovery in patients with incident TB during ART in terms of virologic failure is unknown, and warrants further prospective investigation. Finally, ART effectively decreases TB incidence long term. Our findings highlight the importance of implementing strategies to initiate ART earlier in sub-Saharan Africa to decrease ART-associated active TB.

## Supporting Information

Figure S1 Flowchart of patient selection for analysis. Of all patients initiated on first-line ART, patients with a history of active TB were excluded from the analysis. The remaining patients were followed up for development of incident TB in the first two years after ART initiation. The patients who had not died, were not transferred to another clinic or were lost to follow-up, and had CD4 counts available at 24 months after ART initiation, were selected for comparison of HIV treatment outcomes (box). (TB, tuberculosis; ART, antiretroviral treatment)
(0.25 MB TIF)

Figure S2 Flowchart of post-ART tuberculosis case selection for analysis. The TB drug database was merged with the clinic database to identify post-ART TB cases. Cases coded as having developed TB after ART initiation in both databases were considered as definite TB cases. The TB status of cases identified in only one of the two databases was ascertained by the validation team after review of charts, confirming TB diagnosis and starting date of ART and TB treatment. Unavailability of the chart for review led to exclusion from the analysis. TB in participants occurring after first-line ART initiation were considered as TB cases in the analysis, whether they were diagnosed in our clinic or elsewhere, and whether the diagnosis was based on bacteriological testing or clinical suspicion.
Excluded after chart review: ^a^ no diagnosis of TB (51), TB before ART initiation (64), TB drugs and ART initiated on the same date (2) and chart unavailable for review (65); ^b^ history of previous TB (7); ^c^ chart unavailable for review (7).
(TB, tuberculosis; ART, antiretroviral treatment)
(0.32 MB TIF)

Figure S3 Mortality rates after ART initiation. Mortality rates after ART initiation mirror those of the TB incidence with the highest rates in the first 3–6 months. Point estimates of mortality rates (cases/100pyar, [95% CI]): 0–3 months: 10.05 (8.49–11.89), 3–6 months: 4.76 (3.68–6.15), 6–12 months: 2.89 (2.25–3.71) and 12–24 months: 1.30 (1.05–1.61). (TB, tuberculosis; ART, antiretroviral therapy; CI, confidence intervals; pyar, person years at risk)
(0.64 MB TIF)

Table S1 Baseline characteristics of all patients started on first-line ART. TB, tuberculosis; ART, antiretroviral therapy; IQR, interquartile range; d4T, stavudine; 3TC, lamivudine; NVP, nevirapine; AZT, zidovudine; EFV, efavirenz.
^a^ Data on WHO stage, CD4 count and CD4 percentage were not available for some patients; CD4 counts and percentages were closest recorded values to the baseline start date, maximum 6 months pre-ART.
^b^ Other first line triple ART regimens.
(0.05 MB DOC)

Table S2 HIV treatment outcome after 2 years of ART. HIV treatment outcomes in TB cases were determined for all patients with 24 months of follow-up data available. Outcomes were compared between patients who developed TB in the first 12 months on ART, in months 12–24 on ART and the patients who had remained TB-free after 2 years on ART. To determine effects of TB on CD4 count, suboptimal immune response and immunological failure at 24 months, we restricted our analysis to patients who developed TB within 12 months after ART initiation and to those who remained TB free during the entire 24 months of follow-up to exclude patients on treatment for active TB at the time that CD4 T cell recovery was compared.
^a^ Closest recorded values to 24 months after ART initiation (minimum 21 months and maximum 27 months).
^b^ Data on CD4 count change and CD4 percentage change were not available for 49 and 291 patients, respectively. Suboptimal immune response according to the definition of increase <200 cells/mm^3^ was not determinable in these patients.
^c^ According to the World Health Organization criteria: decrease in CD4 count to pre-ART level or below, decrease in CD4 count from on-treatment peak value by more than 50% or persistent CD4 count <100 cells/mm^3^. ART, antiretroviral treatment; TB, tuberculosis; IQR, interquartile range.
(0.03 MB DOC)
