# Supplementary figures and images for: Incident Tuberculosis during Antiretroviral Therapy Contributes to Suboptimal Immune Reconstitution in a Large Urban HIV Clinic in Sub-Saharan Africa
Source: PLoS One. 2010 May 7;5(5):e10527. doi: 10.1371/journal.pone.0010527 (PMC2866328; doi:10.1371/journal.pone.0010527)

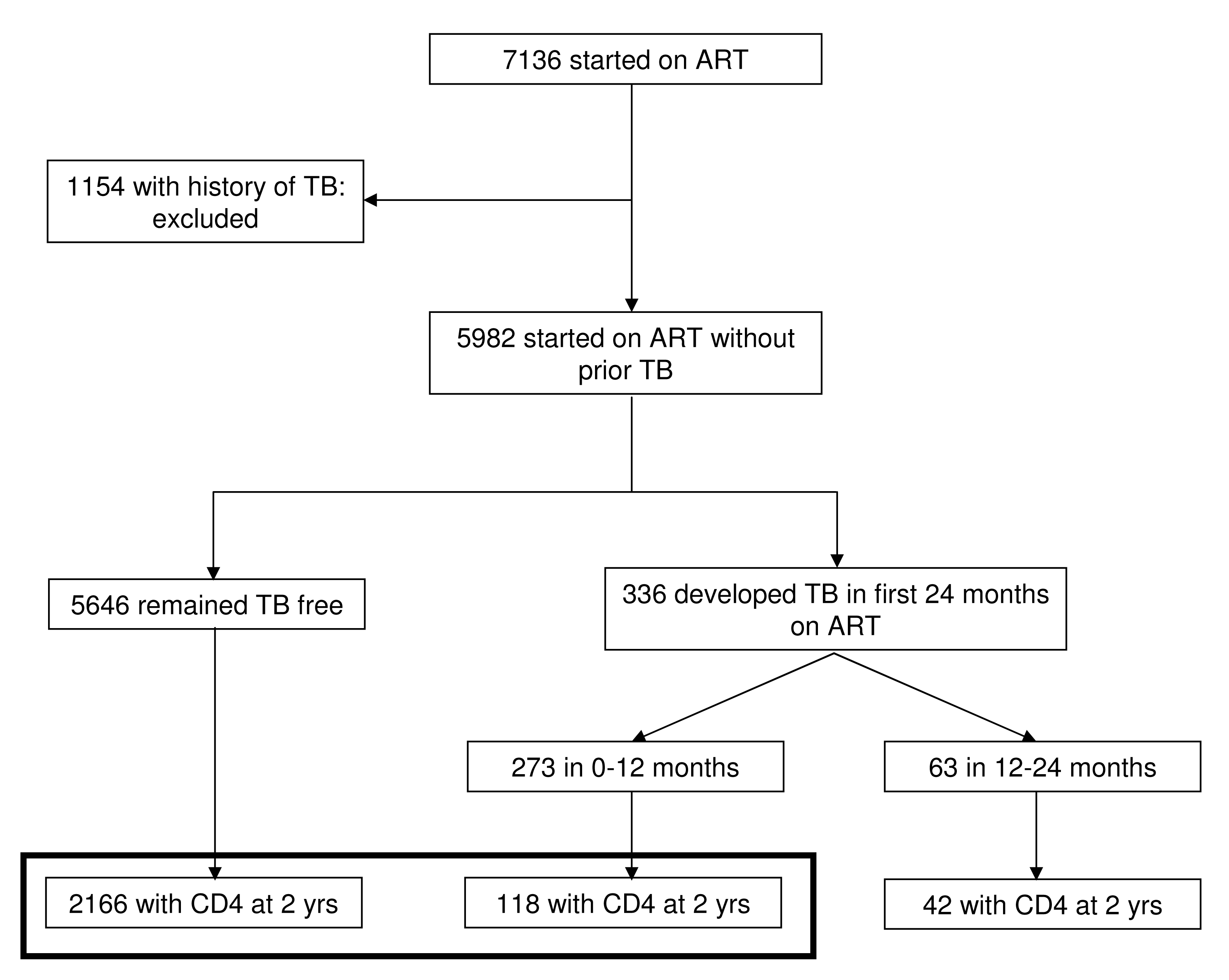

Supplement: Figure S1 — Flowchart of patient selection for analysis. Of all patients initiated on first-line ART, patients with a history of active TB were excluded from the analysis. The remaining patients were followed up for development of incident TB in the first two years after ART initiation. The patients who had not died, were not transferred to another clinic or were lost to follow-up, and had CD4 counts available at 24 months after ART initiation, were selected for comparison of HIV treatment outcomes (box). (TB, tuberculosis; ART, antiretroviral treatment) (0.25 MB TIF) [file pone.0010527.s001.tif]

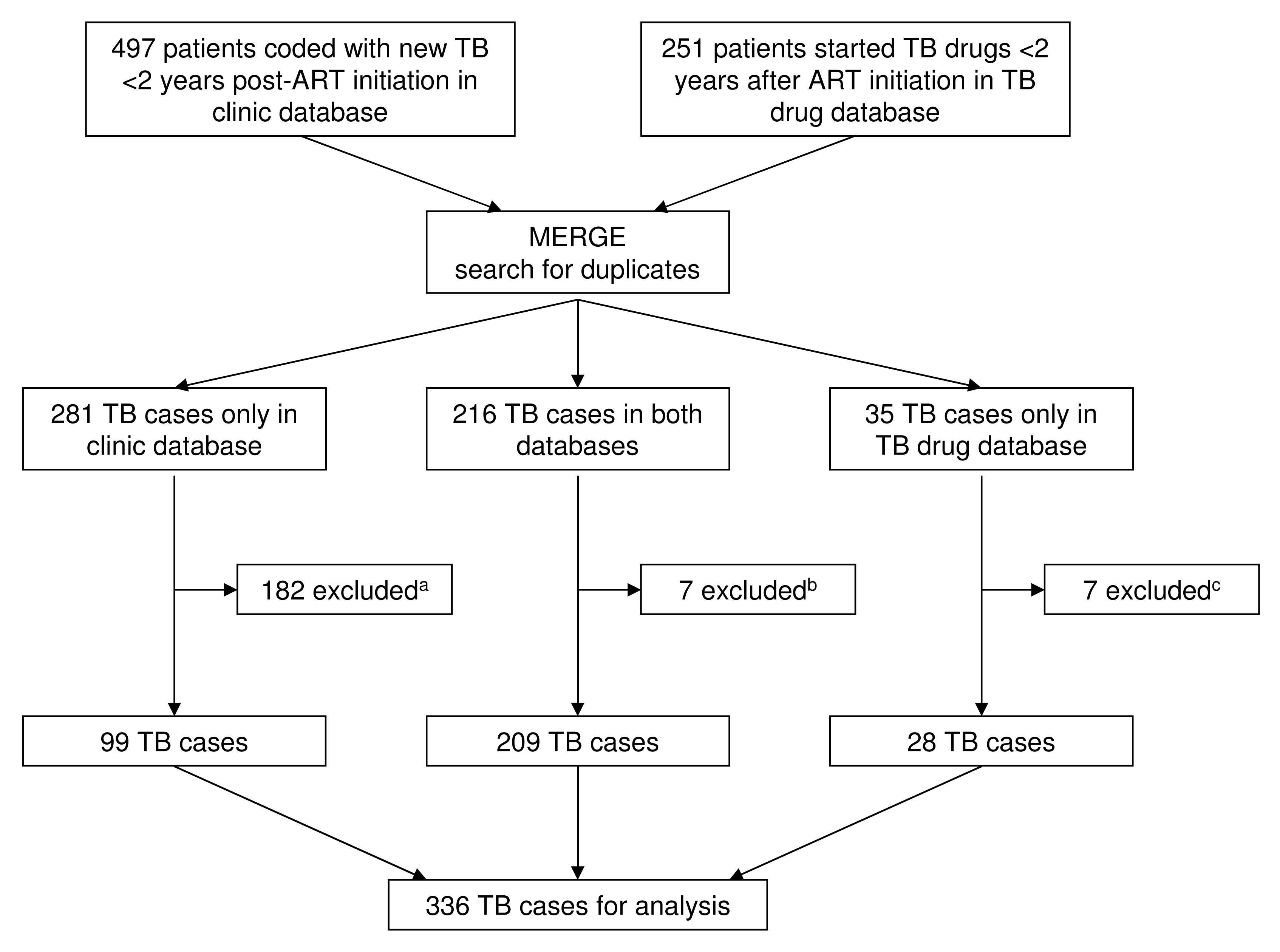

Supplement: Figure S2 — Flowchart of post-ART tuberculosis case selection for analysis. The TB drug database was merged with the clinic database to identify post-ART TB cases. Cases coded as having developed TB after ART initiation in both databases were considered as definite TB cases. The TB status of cases identified in only one of the two databases was ascertained by the validation team after review of charts, confirming TB diagnosis and starting date of ART and TB treatment. Unavailability of the chart for review led to exclusion from the analysis. TB in participants occurring after first-line ART initiation were considered as TB cases in the analysis, whether they were diagnosed in our clinic or elsewhere, and whether the diagnosis was based on bacteriological testing or clinical suspicion. Excluded after chart review: a no diagnosis of TB (51), TB before ART initiation (64), TB drugs and ART initiated on the same date (2) and chart unavailable for review (65); b history of previous TB (7); c chart unavailable for review (7). (TB, tuberculosis; ART, antiretroviral treatment) (0.32 MB TIF) [file pone.0010527.s002.tif]

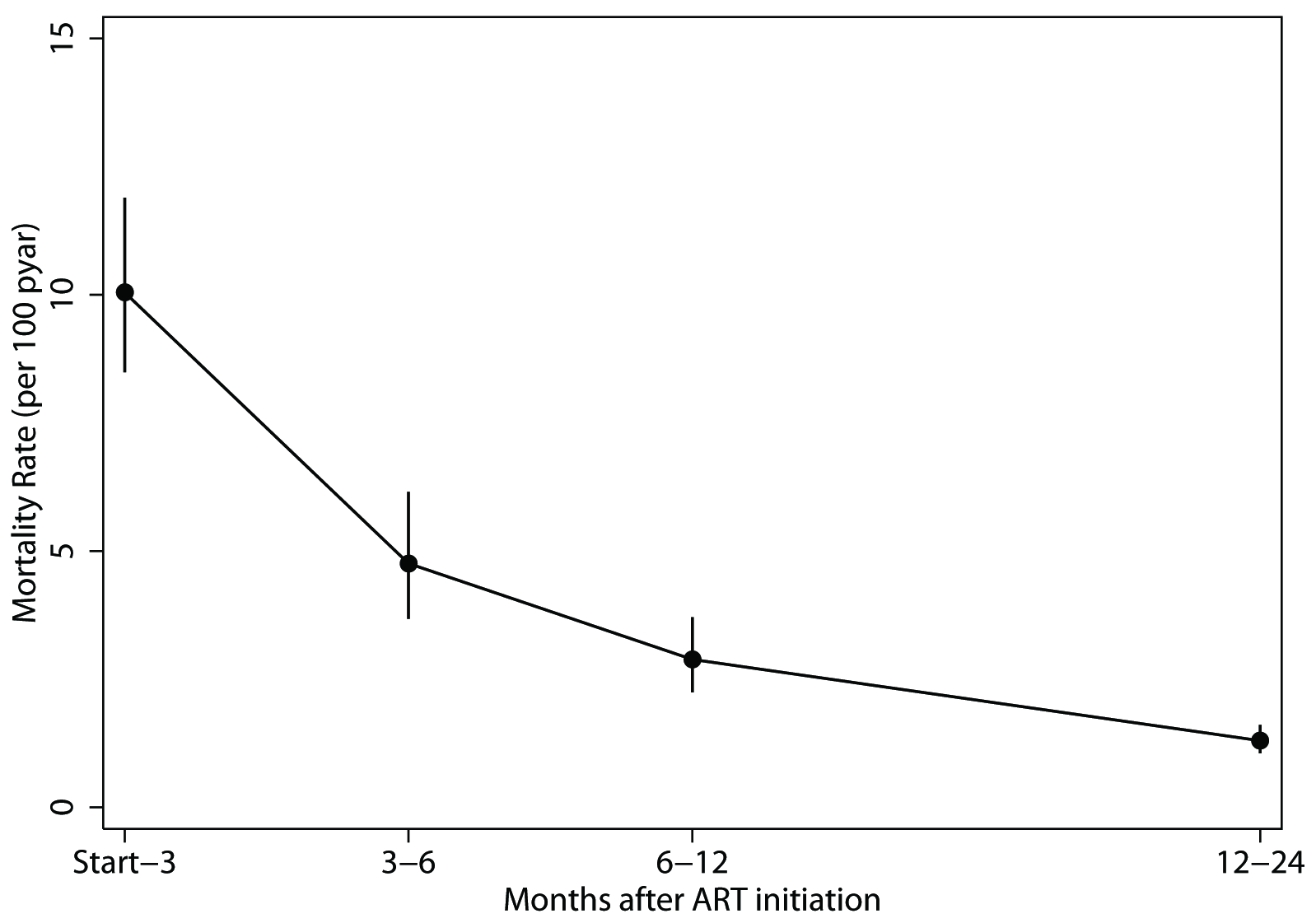

Supplement: Figure S3 — Mortality rates after ART initiation. Mortality rates after ART initiation mirror those of the TB incidence with the highest rates in the first 3–6 months. Point estimates of mortality rates (cases/100pyar, [95% CI]): 0–3 months: 10.05 (8.49–11.89), 3–6 months: 4.76 (3.68–6.15), 6–12 months: 2.89 (2.25–3.71) and 12–24 months: 1.30 (1.05–1.61). (TB, tuberculosis; ART, antiretroviral therapy; CI, confidence intervals; pyar, person years at risk) (0.64 MB TIF) [file pone.0010527.s003.tif]
