# Supplementary material for: Incident Tuberculosis during Antiretroviral Therapy Contributes to Suboptimal Immune Reconstitution in a Large Urban HIV Clinic in Sub-Saharan Africa
Source: PLoS One. 2010 May 7;5(5):e10527. doi: 10.1371/journal.pone.0010527 (PMC2866328; doi:10.1371/journal.pone.0010527)
Supplement: Table S1 — Baseline characteristics of all patients started on first-line ART. TB, tuberculosis; ART, antiretroviral therapy; IQR, interquartile range; d4T, stavudine; 3TC, lamivudine; NVP, nevirapine; AZT, zidovudine; EFV, efavirenz. a Data on WHO stage, CD4 count and CD4 percentage were not available for some patients; CD4 counts and percentages were closest recorded values to the baseline start date, maximum 6 months pre-ART. b Other first line triple ART regimens. (0.05 MB DOC) [file pone.0010527.s004.doc]

| **Category** | **Subcategory** | **All patients (N=7136 [100%])** | **Previous TB cases (excluded) (N=1154 [16.2%])** | **Remaining patients (5982 [100%])** | **TB cases (N=336 [5.6%])** | **Non-TB cases (N=5646 [94.3%])** |
| --- | --- | --- | --- | --- | --- | --- |
| **Sex** (female) |  | 4581 (64.2) | 603 (52.3) | 3978 (66.5) | 185 (55.1) | 3793 (67.2) |
| **Age** (years, mean [SD]) |  | 37 (8.6) | 36.8 (7.8) | 37.2 (8.7) | 36.9 (8.7) | 37.2 (8.7) |
| **WHO stage^a^** | I&II | 2250 (31.6) | 40 (3.5) | 2210 (37.1) | 98 (29.2) | 2112 (37.6) |
|  | III | 2857 (40.2) | 490 (42.5) | 2367 (39.7) | 145 (43.2) | 2222 (39.5) |
|  | IV | 2003 (28.2) | 623 (54.0) | 1380 (23.2) | 93 (27.7) | 1287 (22.9) |
| **Baseline CD4** **count^a^** (cells/mm^3^, median [IQR]) |  | 112 (39, 182) | 73.5 (22, 149) | 117 (42, 182) | 85.5 (31.5, 153.5) | 119 (43, 183) |
| **Baseline CD4** **count^a^** (cells/mm^3^) | 200+ | 1186 (17.5) | 107 (9.6) | 988 (17.3) | 36 (11.3) | 952 (17.7) |
|  | 50-199 | 3620 (53.3) | 554 (49.5) | 3140 (55.1) | 172 (53.8) | 2968 (55.2) |
|  | <50 | 1985 (29.2) | 459 (41.0) | 1572 (27.6) | 112 (35.0) | 1460 (27.1) |
| **Baseline CD4 percentage^a^** (median [IQR]) |  | 7.0 (3.1, 11.1) | 6.0 (2.7, 10.0) | 7.0 (3.3, 11.4) | 6.0 (3.0, 9.0) | 7.0 (3.4, 11.8) |
| **ART regimen** | d4T+3TC+NVP | 3734 (52.3) | 529 (45.8) | 3205 (53.6) | 215 (64.0) | 2990 (53.0) |
|  | d4T+3TC+EFV | 103 (1.4) | 28 (2.4) | 75 (1.3) | 9 (2.7) | 66 (1.2) |
|  | AZT+3TC+NVP | 377 (5.3) | 26 (2.3) | 351 (5.9) | 11 (3.3) | 340 (6.0) |
|  | AZT+3TC+EFV | 2703 (37.9) | 544 (47.1) | 2159 (36.1) | 94 (28.0) | 2065 (36.6) |
|  | Other 1st line ^b^ | 219 (3.1) | 27 (2.3) | 192 (3.2) | 7 (2.1) | 185 (3.3) |
