# Supplementary material for: Incident Tuberculosis during Antiretroviral Therapy Contributes to Suboptimal Immune Reconstitution in a Large Urban HIV Clinic in Sub-Saharan Africa
Source: PLoS One. 2010 May 7;5(5):e10527. doi: 10.1371/journal.pone.0010527 (PMC2866328; doi:10.1371/journal.pone.0010527)
Supplement: Table S2 — HIV treatment outcome after 2 years of ART. HIV treatment outcomes in TB cases were determined for all patients with 24 months of follow-up data available. Outcomes were compared between patients who developed TB in the first 12 months on ART, in months 12–24 on ART and the patients who had remained TB-free after 2 years on ART. To determine effects of TB on CD4 count, suboptimal immune response and immunological failure at 24 months, we restricted our analysis to patients who developed TB within 12 months after ART initiation and to those who remained TB free during the entire 24 months of follow-up to exclude patients on treatment for active TB at the time that CD4 T cell recovery was compared. a Closest recorded values to 24 months after ART initiation (minimum 21 months and maximum 27 months). b Data on CD4 count change and CD4 percentage change were not available for 49 and 291 patients, respectively. Suboptimal immune response according to the definition of increase <200 cells/mm3 was not determinable in these patients. c According to the World Health Organization criteria: decrease in CD4 count to pre-ART level or below, decrease in CD4 count from on-treatment peak value by more than 50% or persistent CD4 count <100 cells/mm3. ART, antiretroviral treatment; TB, tuberculosis; IQR, interquartile range. (0.03 MB DOC) [file pone.0010527.s005.doc]

|  | **All patients (N=2376 [100%])** | **TB cases 0-12 months (N=123 [5.1%])** | **TB cases 12-24 months (N=44 [1.9%])** | **Non-TB cases (N=2209 [93.0%])** |
| --- | --- | --- | --- | --- |
| **CD4 absolute^a^** (cells/mm^3^, median [IQR]) | 310 (216, 426) | 269 (179, 363) | 259 (147, 354) | 312 (221, 431) |
| **CD4 change^a,b^** (cells/mm^3^, median [IQR]) | 205 (121, 304) | 184 (107, 258) | 112 (53, 236) | 208 (123, 309) |
| **CD4 percentage^a^** (median [IQR]) | 17 (13, 22) | 17 (12, 22) | 16 (11, 23) | 17 (13, 22) |
| **CD4 percentage change^a,b^** (median [IQR]) | 11 (7, 14) | 12 (7, 15) | 10 (5, 16) | 10 (7, 14) |
| **Suboptimal immune response^b^** (proportion, <200 cells/mm^3^) | 1117 (48.0) | 67 (56.8) | 29 (67.4) | 1021 (47.1) |
| **Suboptimal immune response** (proportion, not reaching threshold of 200 cells/mm^3^) | 499 (21.0) | 37 (30.1) | 18 (40.9) | 444 (20.1) |
| **Immunological failure^c^** (proportion) | 190 (8.0) | 16 (13.0) | 9 (20.5) | 165 (7.5) |
